# Panorama: a database for the oncogenic evaluation of somatic mutations in pan-cancer

**DOI:** 10.1093/database/baaf086

**Published:** 2026-01-16

**Authors:** Seung-Jin Park, Seon-Young Kim

**Affiliations:** Korea Research Institute of Bioscience and Biotechnology, Daejeon 34131, Republic of Korea; Korea Research Institute of Bioscience and Biotechnology, Daejeon 34131, Republic of Korea; Department of Bioscience, University of Science and Technology (UST), Daejeon 34113, Republic of Korea

## Abstract

Somatic mutations, key alterations in cancer development, exert differential effects across tissues and biological layers, such as transcriptomes, proteomes, and post-translational modifications (PTMs). Although previous pan-cancer studies have characterized the molecular landscape of cancer, the effects of individual somatic mutations across different tissues remain insufficiently explored. Here, we developed Panorama to evaluate the oncogenic potential of single somatic mutations across all cancer types. We collected cancer proteogenomics or multiomics data from over 10 000 individuals across 19 cancer types. Based on five evaluation criteria, we assessed whether a specific mutation affects the abundance of a particular gene’s transcriptome, proteome, or phosphoproteome; the tumor microenvironment; specific RNA- or protein-based signaling pathways; and outlier-level overexpression of PTMs, aiding in potential drug target identification. By leveraging five oncogenic metrics, Panorama quantifies the oncogenic potential of individual somatic mutations and provides a framework for identifying driver mutations by incorporating their downstream effects. With Panorama, researchers can integrate cancer proteogenomics data, providing a comprehensive approach that enhances our understanding of single somatic mutations in specific tissues. Finally, Panorama was developed as a web-based database to ensure easy access for researchers and is freely available at http://139.150.65.64:8080/or https://github.com/prosium/panorama.

## Introduction

Cancer cells, characterized by abnormal proliferation driven by disrupted regulatory mechanisms, are shaped by genomic and epigenomic changes [1, 2]. These alterations act in specific tissues and in ways that depend on biological layers, such as at the DNA, RNA, protein, gene, or pathway levels [3, 4]. The Cancer Genome Atlas (TCGA) studies have enhanced our understanding of the origins and causes of cancer by conducting systematic analyses of genomic changes specific to each cancer type [5–22]. They provided biological insights from multiomics data into precision medicine by depicting the cancer landscape and proposing targeted therapeutic strategies. Several years later, with the launch of the Clinical Proteomic Tumor Analysis Consortium (CPTAC), initiated by advancements in mass spectrometry technology, we now understand the impact of genomic changes on proteins and post-translational modifications (PTMs) in cancer cells [23–39].

Several pan-cancer studies have attempted to harmonize insights from studies on individual cancer types. Using TCGA data, various pan-cancer studies have emerged, including molecular classifications based on cell-of-origin characteristics [4], development of new scores associated with oncogenic differentiation using machine learning [40], identification of oncogenic processes and signaling pathways [3], discovery of driver genes and mutations [41], and perspectives on the immune landscape of cancer [42]. Utilizing CPTAC data, several pan-cancer papers have been published, including studies on the evaluation of oncogenic driver mutations [43], the impact of aberrant methylation patterns [44], the landscape of PTM dysregulation of cancer [45], connections between clinical imaging and proteogenomics [46], the development of data resource package [47], the functional network using machine learning [48], tumor immunity [49], cancer vulnerabilities revealed by whole-genome doubling (WGD) [50], and pan-cancer druggable targets [51]. However, our understanding of the impact of a single somatic mutation across various tissues remains limited, and integrative efforts to analyze consortium-scale data, such as TCGA and CPTAC, remain insufficient.

Therefore, in the current era of cancer proteogenomics, we have integrated data and extended pan-cancer studies to comprehensively evaluate the impact of somatic mutations based on five criteria: (1) association between the mutation and clinical outcomes; (2) dysregulation of gene expression caused by a single somatic mutation at the RNA, protein, and PTMs levels; (3) relationship between the mutation and the tumor microenvironment (TME); (4) dysregulation of signaling pathways induced by the single somatic mutation; and (5) identification of associated therapeutic targets. By integrating data from over 10 000 cancer patients, our curated database evaluates the oncogenic potential of single somatic mutations at the pan-cancer level and identifies putative therapeutic targets for patients harboring specific mutations.

## Methods

### Data collection

**Figure 1.**
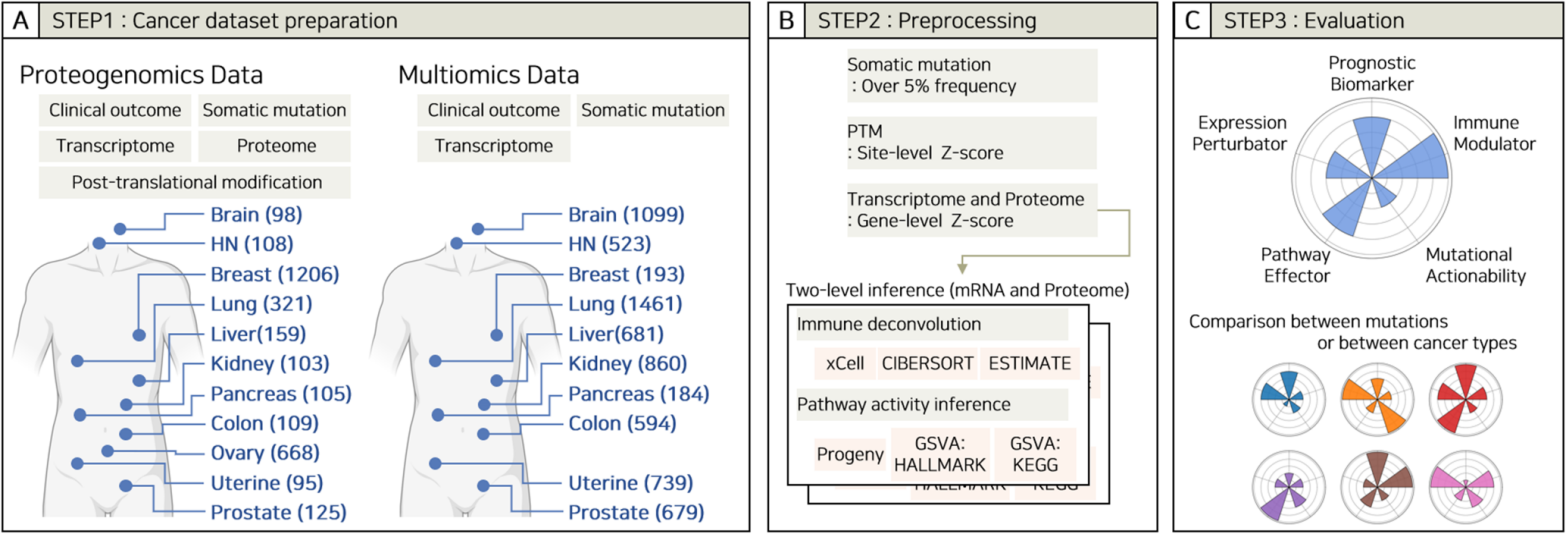
Workflow and scheme for the database. (A) Step 1: Dataset preparations. Data were collected from proteogenomics and multiomics datasets from multiple tissues and biological data types. The number of samples per tissue is shown in parentheses. (B) Step 2: Preprocessing. Data filtering criteria and tools used in the database. (C) Step 3: Evaluation. Five criteria for evaluating a single somatic mutation.

We collected two types of datasets, including proteogenomics and multiomics data (Fig. 1A), derived from several large-scale cancer studies, including TCGA, CPTAC, and Pan-Cancer Analysis of Whole Genomes (PCAWG) (Table S1). For the proteogenomics dataset, data were collected from 11 different cancer types, comprising glioblastoma (GBM) [39], breast carcinoma (BRCA) [21, 31], colon adenocarcinoma (COAD) [25], head and neck squamous cell carcinoma (HNSCC), [32] clear cell renal cell carcinoma [37], hepatocellular carcinoma (HCC) [35], lung adenocarcinoma (LUAD) and lung squamous cell carcinoma (LSCC) [26, 34, 52], ovary cancer (OV), [17, 23] pancreas ductal adenocarcinoma [38] (PDAC), prostate adenocarcinoma [53], and endometrial carcinoma [36]. For multiomics dataset, we retrieved data from 12 different cancer types: lower-grade glioma and GBM [18], COAD [20], HNSCC [19], kidney cancer such as chromophobe renal cell carcinoma, clear cell renal cell carcinoma papillary renal cell carcinoma (KICH, KIRC, and KIRP), [5, 15] LUAD and LUSCC [13, 16, 54, 55], PDAC [56], uterine carcinoma, and uterine corpus endometrial carcinoma (UCEC) [8].

### Data processing

The data obtained in Step 1 were processed by filtering, normalization, and deconvolution (Fig. 1B). Somatic mutations with frequencies >5% within each cohort were selected for analysis. We used processed data directly from public sources (TCGA, CPTAC, and original publications) and subsequently applied Z-score normalization. Specifically, PTM profiles were normalized using site-level Z-scores, whereas transcriptomic and proteomic data were normalized using gene-level Z-scores. Two-level inference was performed using raw normalized data before Z-score calculation based on either mRNA or protein expression. Immune cell-type deconvolution was performed using xCell [57], CIBERSORT [58], and ESTIMATE [59]. For xCell and ESTIMATE, the R wrapper “immunedeconv” [60] was used with default settings. CIBERSORT was implemented using the “CIBERSORT.R” script and the “LM22.txt” reference profile, both obtained from the official CIBERSORT website. Pathway activity inference was performed using Progeny [61] and Gene Set Variation Analysis (GSVA) [62]. For GSVA, the Single Sample Gene Set Enrichment Analysis (ssGSEA) algorithm [63] was employed using the HALLMARK and KEGG gene matrix transposed files retrieved from the official MsigDB [64] website.

Furthermore, our statistical analysis performed intergroup comparisons after the simple deletion of missing data. While this approach is pragmatic for enabling researchers to easily reproduce our analyses and compare them to the original publications, we acknowledge it may introduce statistical bias. However, a sensitivity analysis confirmed this is a rare event, occurring in only 0.35% (*n* = 273) of all tested mutation-gene pairs (*n* = 78 079, *P* < .05, Fisher’s exact test). In future versions of the database, we plan to provide both the raw and imputed versions to offer greater flexibility. The methods described above (xCell, CIBERSORT, ESTIMATE, Progeny, and GSVA) are standard, well-validated methodologies widely used in pan-cancer analyses [43–45, 49, 65, 66]. We employed default settings as recommended by the tool developers, which align with these established protocols and ensure comparability and reproducibility with previous studies. Applying immune deconvolution tools to proteomic data is an important and emerging field, though a single “gold-standard” method is still in development. However, a recent study by Song *et al*. [67], while noting the lack of a gold-standard dataset, reported that xCell provides the most robust results when comparing proteome-based predictions to RNA-based benchmarks. Therefore, Panorama provides results from xCell, ESTIMATE, and CIBERSORT to allow for a comprehensive comparison of these methods.

### Oncogenicity evaluation

Each mutation was evaluated across five categories: prognosis, abnormal gene expression, TME, pathway activity, and actionability (Fig. 1C). (1) Prognostic biomarker (PB): Prognostic significance was determined if, in any of the following, overall survival, disease-specific survival, disease-free survival, progression-free survival, or relapse-free survival, patients with the mutation had poorer outcomes than those without the mutation. Statistical significance was evaluated using log-rank tests. (2–3) Expression perturbator (EP) and immune modulator (IM): The impact of a mutation on the expression of genes or immune cell populations was evaluated by counting the number of genes or immune cell populations that were relatively increased in the mutation group compared to those in the wild-type (WT) group, with a false discovery rate (FDR) based on Student’s *t*-test. (4) Pathway effectors (PE): Pathways that exhibited statistically significant increases or decreases in intensity associated with a given mutation compared to WT patients were evaluated. The criterion for significance was FDR based on Student’s *t*-test. (5) Actionable driver (AD): Identification of therapeutic targets involved in discovering phosphorylation events upregulated to outlier levels relative to changes in RNA expression driven by a somatic mutation.

### Comparison method for TP53 mutations at the pan-cancer level

For the PB metric, *P*-values were transformed using log10. For MA, we calculated the interquartile range (IQR) of the PTM data and defined PTMs exceeding QI + 1.5 × IQR as upper outliers; the number of such PTMs was then normalized by the total PTM count. For EP, the count of genes with a SignedFDR ≥ 1.301 (indicating positive significance) was normalized by the total number of genes. Similarly, for IM and PE, the number of terms with a SignedFDR ≥ 1.301 was normalized by the total number of terms. Each mutation was then ranked as follows: the top two cancer types received five points, the following two types received four points, the following four types received three points, the subsequent two types received two points, and the final two types received one point. Additionally, if a mutation lacked a term or gene with a SignedFDR ≥ 1.301, it was assigned a minimum score of 1.

### Driver mutations

Mutations with frequencies of at least 5% were selected for each cohort. For every mutation, we calculated a score for each metric by dividing the number of values with a SignedFDR ≥ 1.301 by the total number of values. For PB metric, we applied the -log10 transformation to the *P*-values. This process yielded five scores for each mutation. Subsequently, we performed Z-score normalization for each cohort using these five metrics and computed their average, which was defined as the average oncogenic score (AOS). Finally, to distinguish driver mutations from passenger mutations, we performed a robust outlier analysis on the AOS values for each cohort. This analysis employed two simultaneous, standard statistical methods: (1) an IQR method, where outliers were defined as any value exceeding Q3 + 1.5 × IQR, and (2) a Z-score method. For the Z-score method, we defined outliers as mutations with a |Z-score| > 2.5. This threshold was chosen because it is stricter than ± 2SD (which captures ∼95% of the data) and is statistically grounded, identifying approximately the top 1% of outliers. Based on the results of these two tests and a curated list of known driver genes [41], we classified all mutations into a four-tier grading system to systematically assess confidence, as shown in Fig. 4(B): Grade 1 (identified as an outlier by both tests + known driver), Grade 2A (identified by both tests + novel candidate), Grade 2B (identified by one test + known driver), and Grade 3 (identified by one test + novel candidate).

### Database construction and web interface

All the raw data were obtained from publicly available sources (Table S1). Additionally, the processed data derived from the raw data are shared in the Panorama GitHub repository (https://github.com/prosium/panorama). Furthermore, for interactive web applications, Panorama was constructed using data science libraries, such as Pandas, NumPy, Plotly, SciPy, and Streamlit. Furthermore, to support bioinformaticians in conducting large-scale analyses locally or in parallel, we have provided all Panorama results in the Parquet format on GitHub.

**Figure 2.**
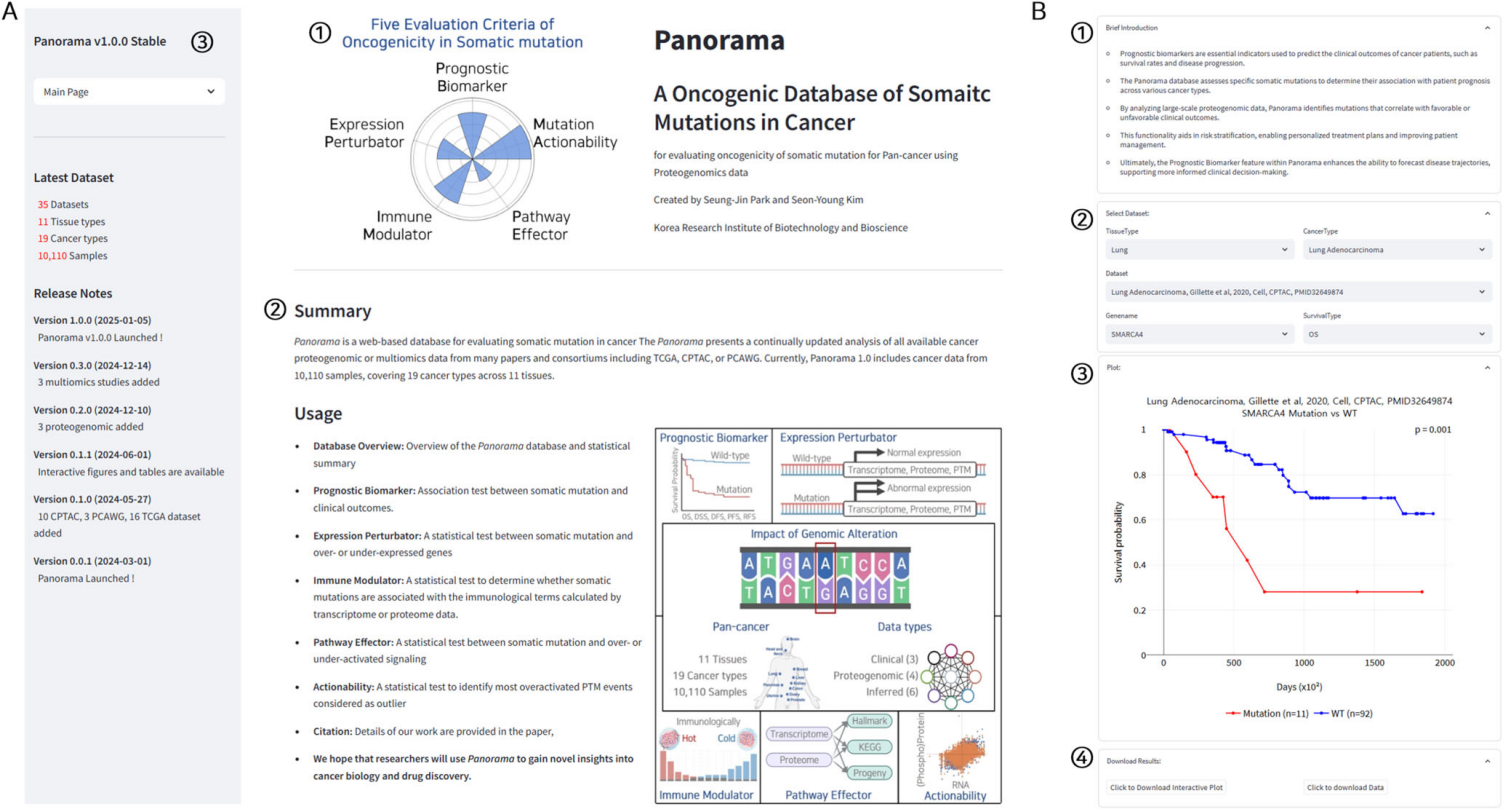
The web interface of Panorama. (A) Overview of the Panorama web interface, (1) logo of the Panorama database, (2) a brief description and functionalities of the Panorama database, and (3) Panorama menu bar. (B) Detailed view of the Panorama database function interface: (1) a brief description of the function, (2) dataset selection options including tissue type, cancer type, dataset, gene name, and survival type, (3) result plot, and (4) download buttons for the plot and underlying data.

The Panorama web interface is organized with a logo, a database summary, and brief descriptions of the five functions on the right, while the left side features a menu bar, an overview of cancer data, and release notes (Fig. 2A). Clicking on the menu reveals several options, including the main page (homepage), a database overview (detailed data status), and five functions. Selecting one of the five functions allowed the user to navigate to a page with a brief introduction, dataset selection, result plots, and a download menu (Fig. 2B). This uniform layout across all five functions ensures consistency and enhances usability.

### Database application

We describe several use cases of the Panorama database below to demonstrate its functionality.

## SMARCA4 mutation as a PB in LUAD

**Figure 3.**
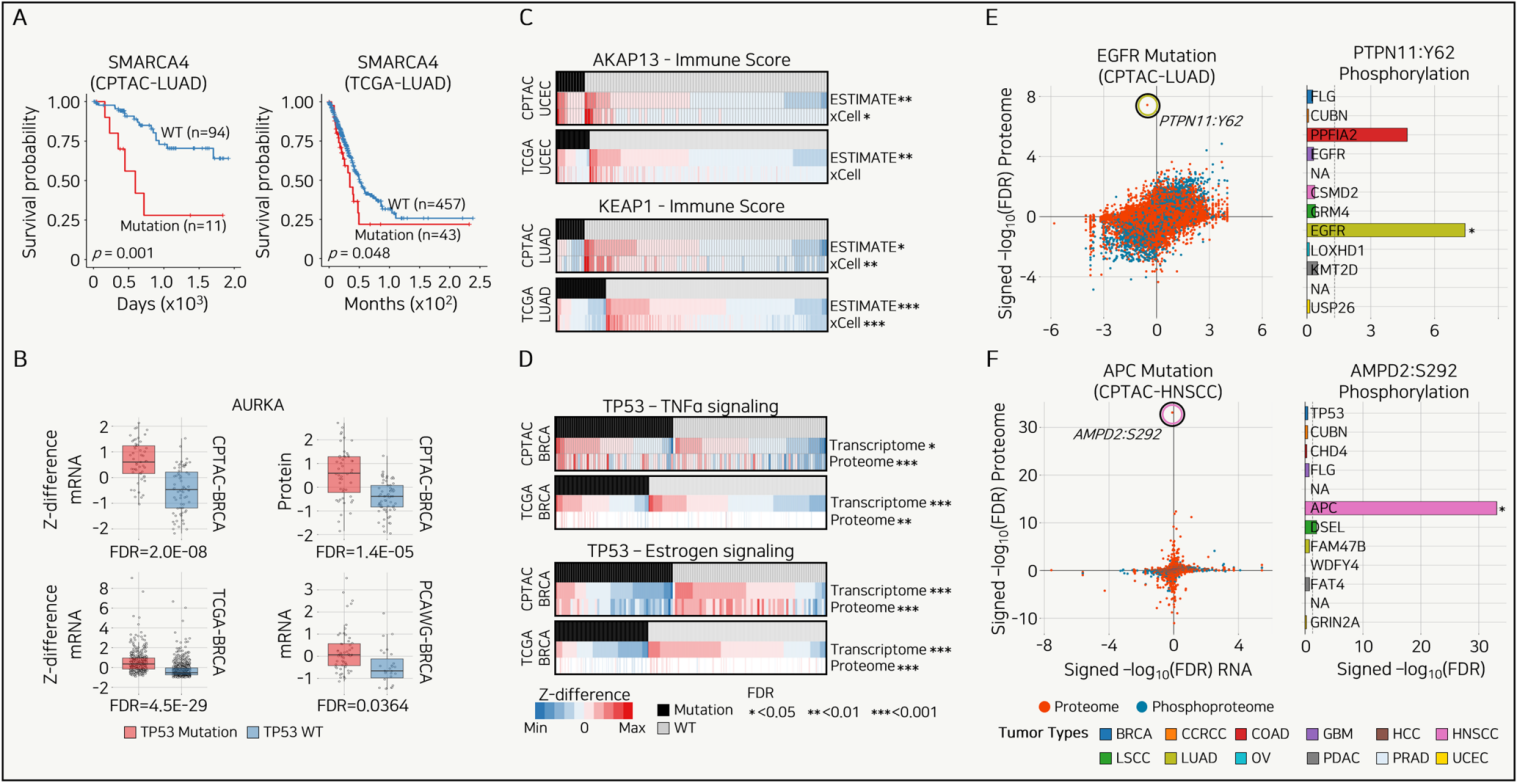
Use cases of Panorama. (A) Kaplan–Meier survival curves for SMARCA4 mutations in LUAD from CPTAC (left) and TCGA (right) datasets. Log-rank test. (B) Box plots comparing Z-differences in mRNA and protein expression levels for AURKA across multiple breast cancer (BRCA) cohorts. *P*-value is calculated from the Student’s *t*-test and converted to FDR using the Benjamini–Hochberg method. (C) Heatmaps illustrating the relationship between immune scores from ESTIMATE and xCell and gene expression changes as Z-differences, with significant associations marked by FDR thresholds. The *P*-value is calculated from the Student’s *t*-test and converted to FDR using the Benjamini–Hochberg method. (D) Heatmaps showing the association between mutation status and tumor necrosis factor-alpha (TNFα) and estrogen signaling pathways in BRCA. Expression changes are represented as Z-differences, with significant differences denoted by asterisks indicating FDR thresholds. The *P*-value is calculated from the Student’s *t*-test and converted to FDR using the Benjamini–Hochberg method. (E and F) (left) Scatter plots showing the correlation between RNA, proteome, or PTM changes for mutations in two tumor types, with outlier phosphorylation sites highlighted. The right panels display bar plots comparing the signed -log10(FDR) for proteome phosphorylation targets across all tumor types. *P*-value is calculated from the Student’s *t*-test and converted to FDR using the Benjamini–Hochberg method.

Mutations can promote tumor growth, proliferation, and metastatic potential and reduce treatment responsiveness. These effects may be associated with improved or worsened patient survival rates. When the effect of a specific mutation on the prognosis is consistently observed, it serves as a PB. In the CPTAC-LUAD cohort, 11 patients with SMARCA4 mutations showed a significantly poorer prognosis than the 94 WT patients (*P* = .001) (Fig. 3A). Similarly, in the TCGA-LUAD cohort, 43 patients with SMARCA4 mutations had significantly poorer outcomes than 457 WT patients (*P* = .048) (Fig. 3A). Given that patients with the SMARCA4 mutation in LUAD consistently exhibit a poor prognosis across two independent cohorts, SMARCA4 can be used as a PB for LUAD, as previously reported [68].

## TP53 mutation as an EP in BRCA

One approach to identifying factors that play critical roles in cancer development and progression is to identify genes specifically upregulated or overexpressed in cancer cells, as these are mutation-specific oncogenes that promote tumor formation. We hypothesized that if a gene is upregulated in patients with a mutation, and this upregulation is consistently observed across independent cohorts, the overexpressed gene is not only a target of the specific mutation but also drives cancer development in the context of that mutation.

In the CPTAC-, TCGA-, and PCAWG-BRCA cohorts, the frequently observed TP53 mutation group showed consistent and significant overexpression of AURKA compared to the TP53 WT group at both the transcriptomic and proteomic levels (Fig. 3B). Consistent upregulation of AURKA in association with TP53 mutations provides strong evidence that AURKA plays a critical role in cancer development or progression in the context of TP53 mutation [69].

## AKAP13 and KEAP1 mutations as IMs in UCEC and BRCA

Immunotherapy, increasingly used in cancer treatment, is more effective when there is a high infiltration of immune cells, including T cells, in the TME, a condition referred to as an immunologically “hot” state. In contrast, tumors in an immunologically “cold” state, characterized by fewer or suppressed immune cells, are less likely to respond to immunotherapy. We analysed mutations associated with immunologically hot or cold states using immune scores derived from ESTIMATE and xCell.

In the CPTAC- and TCGA-UCEC cohorts, the AKAP13 mutation was associated with elevated immune scores across both tools and independent cohorts (Fig. 3C, top). Conversely, the KEAP1 mutation in the CPTAC- and TCGA-LUAD cohorts was associated with a decreased immune score in both the tools and independent cohorts (Fig. 3C, bottom). The consistent patterns suggest that AKAP13 and KEAP1 mutations can be proposed as immunotherapy markers, as they significantly alter the TME to a hot or cold state, as is well known. These findings can enhance our understanding of immune evasion mechanisms, improve the prediction of immunotherapy responses by cancer type, and provide opportunities to identify new immunotherapeutic targets [70, 71].

## TP53 mutation as a PE in BRCA

Measuring activation at the pathway level rather than the expression of a single gene is essential for a systematic understanding of biological mechanisms. We utilized the hallmark gene set and applied GSVA to calculate ssGSEA scores, comparing patients with specific mutations to those with WT. In the CPTAC- and TCGA-BRCA cohorts, patients with TP53 mutations showed significantly increased tumor necrosis factor-alpha (TNFα) signaling at both the transcriptome and proteome levels (Fig. 3D, top). Conversely, TP53 mutations in CPTAC- and TCGA-BRCA cohorts were associated with a significant decrease in estrogen signaling (Fig. 3D, bottom).

The consistent increase in TNFα signaling associated with TP53 mutations suggests that TP53 mutations significantly alter gene expression and signaling pathways. Furthermore, it suggests TNFα signaling as a potential therapeutic target for breast cancer patients harboring TP53 mutations [72].

## EGFR and APC mutations as therapeutic targets in LUAD and HNSCC

A significant increase in phosphorylation to outlier levels in patients with specific mutations suggests abnormal activation of the corresponding signaling pathway or protein, indicating its potentially critical role in cancer development or progression. We identified statistically significant phosphorylation events at the outlier level in patients with specific mutations and validated their unique significance across 12 tumor types.

In LUAD, patients with EGFR mutations showed a significantly higher FDR value for Y62 phosphorylation of PTPN11 than for the next-highest phosphorylation, K269, of BLVRA (Fig. 3E, left). At the pan-cancer level, various mutations, including those in FLG, CUBN, and PPFIA2, significantly increased Y62 phosphorylation of PTPN11; however, none of these mutations elevated Y62 phosphorylation of PTPN11 to an outlier level (Fig. 3E, right). In HNSCC, S292 phosphorylation of AMPD2 was observed in patients with APC mutations, and this phosphorylation was the only significantly different event at the outlier level (Fig. 3F). The finding that PTPN11 is a well-established strong potential therapeutic target in LUAD and AMPD2 is a novel potential therapeutic target in HNSCC, despite showing no significant differences at the transcriptomic or proteomic levels (FDR > 0.05), emphasizes the importance of proteogenomic data [34].

## Tissue-dependent dynamics of TP53 mutations

**Figure 4.**
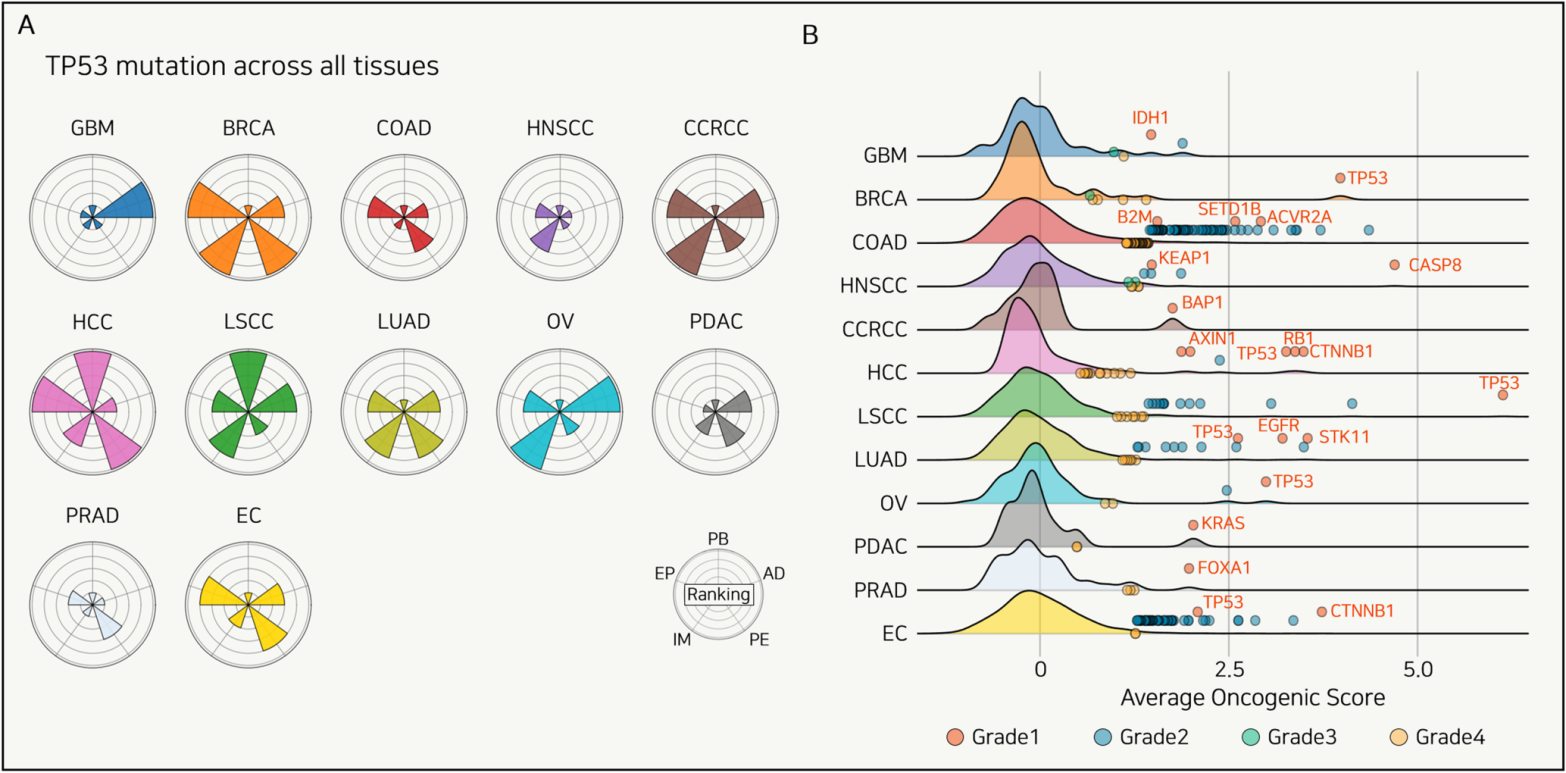
Tissue-specificity of TP53 mutation and candidate driver mutations. (A) Radar plots displaying the evaluation of TP53 mutations across 12 cancer types. Each sector of the radar plot represents a different functional category: PB, EP, IM, PE, and AD. (B) Distribution of the AOS across 12 cancer types. The density plots show the complete distribution of AOS values for all mutations with >5% frequency in each cohort. The overlaid dots represent only those mutations identified as statistical outliers and classified into Grades 1, 2A, 2B, and 3. This four-tier grading system classifies mutations based on their outlier status and prior driver knowledge, as detailed in the section “Methods.” Key Grade 1 driver mutations are labeled with their gene names.

Even when an identical TP53 mutation occurs at the same locus within the gene, the functional consequences and impact on tumorigenesis are highly dependent on the tissue context [73]. As shown in Fig. 4(A), we compared the five TP53 mutation functions across various tissues at the pan-cancer level.

In HCC, TP53 mutations are a poor-prognosis biomarker, whereas in LSCC, they are a better-prognosis biomarker; no significant association was observed in the other 10 cancer types. Additionally, more genes showed increased expression in BRCA and HCC compared to other cancers, and immune cell populations were notably elevated in BRCA and COAD. Moreover, HCC and BRCA showed greater upregulation of signaling pathways than other cancer types, while GBM and OV showed a greater number of PTMs reaching outlier levels. Using Panorama to analyse TP53 mutations at the pan-cancer level enables the identification of therapeutic targets and prognostic evaluation for specific cancer types, thereby uncovering tissue-dependent heterogeneity in TP53 mutation mechanisms.

## Driver mutation discovery

Identifying driver mutations in cancer data is challenging. Most tools select driver mutations based on factors such as mutation recurrence across the genome, their localization within functional domains, and the surrounding context [74]. We assumed that driver mutations would be associated with a greater influence on prognosis, a higher number of overexpressed genes, overactivated immune cell populations, hyperactivated signaling pathways, and more actionable PTM targets than passenger mutations (see the section “Methods”). Therefore, we calculated the average values of the five Panorama-measured factors for all mutations with a frequency of at least 5% across the 12 tissue types.

At the pan-cancer level, we identified upper outliers (displayed in blue) based on each mutation’s AOS and highlighted the known driver mutations for each cancer type in red (Fig. 4B). Across all 12 cancer types, we divided the known driver mutations into novel candidates. We identified at least one known driver mutation for each cancer type and proposed several novel mutations (Table S2). Notably, although not previously reported, the SLC8A3 mutation in LUAD was associated with significant increases in the immune score, estimate score, and stroma score among patients harboring the mutation. Unlike many existing tools for driver mutation discovery, our approach using AOS enables the detection of novel mutations. Through this approach, we propose a novel method for identifying driver mutations using cancer proteogenomic data that comprehensively considers both mutation frequency and downstream mutation effects.

## Discussion and perspectives

In Panorama, we aimed to elucidate the diverse impacts of single somatic mutations on cancer by integrating proteogenomic data, including clinical data, from over 10 000 patients with various cancer types. We performed robust analysis to highlight the intricate biological consequences of specific mutations. Over the years, numerous web-based databases have been developed for comprehensive pan-cancer analyses. These include tools that focus on comparisons between normal and tumor tissues [75], broad analysis platforms in cancer genomics [76], and extensive association analysis tools that leverage proteogenomic data [77]. Panorama’s unique contribution is its mutation-centric group comparison framework. It provides a single, streamlined visualization that allows for a simultaneous comparison of how a single somatic mutation (versus the WT group) affects three layers—RNA, proteome, and phosphoproteome. This function is a core feature of Panorama, as it intuitively reveals how a genomic alteration may bypass the transcriptome and directly enact its function at the proteome or phosphoproteome level, a specific question not directly streamlined by other correlation-based platforms. Furthermore, the ability to analyse and visualize associations with derived data, such as immune signaling or signaling activity, in a single heatmap is a unique advantage of Panorama.

Various genomic alterations, in addition to single somatic mutations—such as copy number or structural changes, WGD, fusions, and aberrant methylation—can underlie the changes observed in our PB, EP, IM, PE, and AD metrics. In particular, the recent study by Chang *et al*. [50] demonstrated that WGD does not act uniformly but can be classified into different types based on the level of genomic instability, which varies in a cancer-type-specific manner. Crucially, they found that these WGD types are distinctly associated with specific “kinase phosphorylation networks” and cell cycle pathway activation. This finding suggests that large-scale genomic events like WGD may ultimately converge on specific phosphorylation pathways to drive tumorigenesis, highlighting the necessity of evaluating WGD as a potential driver of the “Actionable Driver (AD)” events identified in Panorama. Therefore, we plan to expand Panorama to include various other genomic events, such as WGD, to provide a more comprehensive functional analysis and will also continuously integrate new data from publicly available databases.

Furthermore, co-occurring or mutually exclusive mutations can act as confounding factors when analysing the impact of a single mutation [78, 79]. Panorama provides lists of co-occurring and mutually exclusive mutations across all cohorts to mitigate this issue, enabling a more accurate interpretation of the results (Table S3). Recently, it was shown that even when the same EGFR amplification is found in recurrent GBM, treatment resistance arises from decreased EGFR activation and increased BRAF activation at the protein and phosphorylation levels [80]. Panorama will not be limited to primary cancer, and we plan to add functionality to compare “sensitive vs. resistant” functions. Additionally, among the newly identified subtypes of nonsmall cell lung cancer, some have been defined by phosphorylation features and others by immune scores, which were then in turn used to predict therapeutic response [81]. We are planning future research to move beyond single-mutation analysis and define new subtypes based on the AOS, which reflects all five features of Panorama.

Our comprehensive proteogenomic database, Panorama, integrates analyses from numerous studies to advance our understanding of how single somatic mutations differentially affect cancer progression across diverse tissues. By integrating diverse data types, we identified key PBs, elucidated the mechanisms of immune modulation, and identified potentially ADs. This approach enables precise characterization of somatic mutation functions, facilitates a deeper understanding of cancer heterogeneity, and enhances patient treatment efficacy. Furthermore, it is provided as a web-based database to ensure easy access for researchers.

## Supplementary Material

baaf086_Supplemental_Files

## Data Availability

Database files in Parquet format and code are freely available for download on GitHub (https://github.com/prosium/panorama).
